# A Phenotypic Analysis of Involucrin–Membrane-Bound Ovalbumin Mice after Adoptive Transfer of Ovalbumin-Specific CD8^+^ T Cells

**DOI:** 10.1016/j.xjidi.2022.100127

**Published:** 2022-03-30

**Authors:** Yujin Nakagawa, Gyohei Egawa, Toshiya Miyake, Saeko Nakajima, Atsushi Otsuka, Takashi Nomura, Akihiko Kitoh, Teruki Dainichi, Jun-ichi Sakabe, Akihiko Shibaki, Yoshiki Tokura, Tetsuya Honda, Kenji Kabashima

**Affiliations:** 1 Department of Dermatology, Kyoto University Graduate School of Medicine, Kyoto, Japan; 2 Singapore Immunology Network (SIgN) and Skin Research Institute of Singapore (SRIS), Agency for Science, Technology and Research (A∗STAR), Singapore, Singapore; 3 Shibaki Dermatology Clinic, Sapporo, Japan; 4 Department of Dermatology, Hamamatsu University School of Medicine, Hamamatsu, Japan

**Keywords:** CTL, cytotoxic T lymphocyte, DC, dendritic cell, GVHD, graft-versus-host disease, Ivl, involucrin, K14, keratin 14, K5, keratin 5, KC, keratinocyte, LC, Langerhans cell, LN, lymph node, mOVA, membrane-bound ovalbumin, OVA, ovalbumin, Tg, transgenic, Treg, regulatory T cell

## Abstract

To investigate the mechanism of autoimmunity and peripheral tolerance in the skin, several transgenic mouse strains expressing membrane-bound ovalbumin (mOVA) as an epidermal self-antigen under the control of keratinocyte-specific promotors, such as keratin 5 and keratin 14, were employed in combination with adoptive transfer of CD8^+^ T cells from OT-I mice (OT-I T cells) that recognize an ovalbumin-derived peptide. However, these strains showed bodyweight loss and required additional inflammatory stimuli, such as γ-irradiation and tape-stripping, to induce skin inflammation. In this study, we generated a mouse strain expressing mOVA under the control of human involucrin promoter (involucrin-mOVA mice). In contrast to previous strains, involucrin-mOVA mice spontaneously developed skin inflammation after the transfer of OT-I T cells in the absence of external stimuli without significant bodyweight loss. We focused on the skin infiltration process of OT-I T cells and found that transferred OT-I T cells accumulated around the hair follicles in the early phase of skin inflammation, and in the later phase, the skin inflammation spontaneously resolved despite the remaining OT-I T cells in the skin. Our involucrin-mOVA mice will provide a promising tool to investigate the pathogenesis and the tolerance mechanisms of cytotoxic skin autoimmunity.

## Introduction

Graft-versus-host disease (GVHD) is representative skin disorder caused by a direct attack on the epidermal keratinocytes (KCs) by CD8^+^ cytotoxic T lymphocytes (CTLs) (Okiyama and Fujimoto, 2015). Allogeneic CTLs are responsible for the pathogenesis of GVHD. It is well known that the interaction between CTLs and KCs is essential for the initiation of KC necrosis; however, the in vivo dynamics of each immunological process in these diseases have not been fully elucidated. It remains unknown when and where CTLs enter the epidermis (from the dermis) to initiate skin inflammation, and when and how this inflammation is terminated subsequently.

To investigate these issues, several transgenic (Tg) mouse strains that expressed membrane-bound ovalbumin (mOVA) as a self-antigen under the control of epidermis-specific promoters, such as keratin 5 (K5) and keratin 14 (K14), were generated; one strain of K5-mOVA mice and four strains of K14-mOVA mice. In combination with adoptive transfer of CD8^+^ T cells from OT-I mice (OT-I T cells), which recognize an ovalbumin (OVA)-derived peptide in the context of H-2K^b^ (Hogquist et al., 1994), these strains have been used as valuable tools of a cutaneous GVHD model to investigate the pathogenesis of skin autoimmunity and peripheral tolerance (Miyagawa et al., 2010); however, each strain has several limitations to study these questions. For instance, a sublethal irradiation or tape-stripping was necessary for the induction of OVA-specific skin inflammation in K5-mOVA mice and in one K14-mOVA strain, respectively (Azukizawa et al., 2003; Bianchi et al., 2009). Another K14-mOVA strain did not exhibit skin inflammation even after the transfer of OT-I T cells (Miyagawa et al., 2008). Although a strain of K14-mOVA mice spontaneously developed OVA-specific skin inflammation, they also exhibited apparent bodyweight loss, which was presumably caused by the loss of food intake owing to submucosal inflammation in the esophagus (Shibaki et al., 2004). In another K14-mOVA strain, transferred OT-I T cells fell into apoptosis in skin-draining lymph nodes (LNs) and no skin phenotype was induced (Bursch et al., 2009). Thus, the establishment of a mouse model that spontaneously develops skin inflammation after the OT-I T-cell transfer without marked mucosal involvement has been demanded.

In this study, we generated a Tg strain that expressed mOVA under the control of human involucrin (Ivl) promoter, Ivl-mOVA mice. Ivl is synthesized in epidermal KCs of the stratum spinosum and is one of the constituent proteins of the cornified cell envelope. Using this strain, we observed the initiation and the termination processes of cytotoxic immune reactions to the epidermis. We found that transferred OT-I T cells spontaneously expanded in the skin-draining LNs and that they accumulated around the hair follicles during the initiation phase of skin inflammation, which finally led to GVHD-like skin lesion without significant bodyweight loss. This skin inflammation spontaneously resolved despite remaining OT-I T cells in the skin with increasing skin-infiltrated regulatory T cells (Tregs). We depleted Tregs in Ivl-mOVA mice and found that the transfer of a small number (1 × 10^5^) of OT-I T cells could induce skin lesion only in Treg-depleted mice. Because follicular involvement is considered to be a suggestive finding of acute cutaneous GVHD in humans, our mouse model may share some pathological mechanisms with that disease.

## Results

### Ivl-mOVA mice express OVA as an epidermal self-antigen

**Figure 1:**
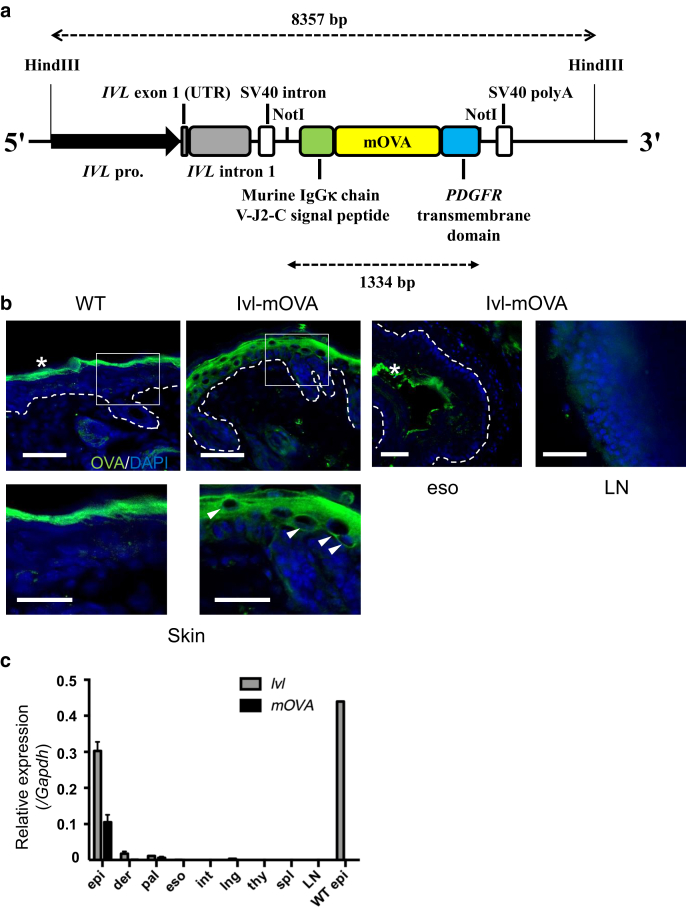
**Ivl-mOVA mice express OVA as an epidermal self-antigen.** (**a**) Construction of *mOVA* transgenic vector and generation of *mOVA*^*Tg*^ mice. (**b**) Immunohistochemistry of OVA expression in the epidermis. Alexa Fluor 488-positive signals in the dermis are considered to be nonspecific. Note the OVA expression on cell membrane of epidermal keratinocytes in Ivl-mOVA mice, but not in wild-type mice (arrowheads). Green, OVA; Blue, DAPI; and ∗, nonspecific signal from the stratum corneum of the skin and keratin layer of esophageal epithelium. Bar = 50 μm. Lower panels are higher magnification views of each square. Bar = 25 μm. (**c**) Quantitative RT-PCR of *Ivl* and *mOVA* mRNA expressions in each tissue. Each tissue (n = 3 mice) except for WT epi (n = 1 mouse). bp, base pair; der, dermis; epi, epidermis; eso, esophagus; int, intestine; Ivl, involucrin; LN, lymph node; lng, lung; mOVA, membrane-bound ovalbumin; OVA, ovalbumin; pal, palate; polyA, polyadenylation site; pro., promoter; spl, spleen; thy, thymus; UTR, untranslated region; WT, wild type.

We generated a Tg strain using a human Ivl promoter vector and a p*K14-mOVA* vector. Ivl-mOVA mice expressed mOVA under the control of human Ivl promoter (Figure 1a). These Tg mice presented a similar appearance and growth to that of wild-type littermates (data not shown). To confirm the expression of mOVA limited to the epidermis, we examined OVA expression by immunohistochemistry. Consistent with Ivl being a marker of differentiating KCs, the OVA expression was detected throughout the epidermis, whereas the expression at the basal layer was substantially weak (Figure 1b). To check mOVA expression in other tissues, we harvested mRNA from various epithelial tissues and lymphoid organs such as the epidermis, dermis, palate, esophagus, intestine, lung, thymus, spleen, and skin-draining inguinal LN (Figure 1c). Quantitative RT-PCR analysis revealed that mOVA was highly expressed in the epidermis. A marginal expression of mOVA was detected in the palate but not in other epithelial tissues. No mOVA expression was detected in the primary and secondary lymphoid tissues such as the thymus, spleen, and inguinal LN. The absence of OVA expression in the esophagus and the lymph node was also confirmed by immunohistochemistry (Figure 1b). These results indicate that mOVA is predominantly expressed in the epidermis of Ivl-mOVA mice.

### Transferred OT-I T cells spontaneously expanded in the skin-draining LNs in Ivl-mOVA mice

**Figure 2:**
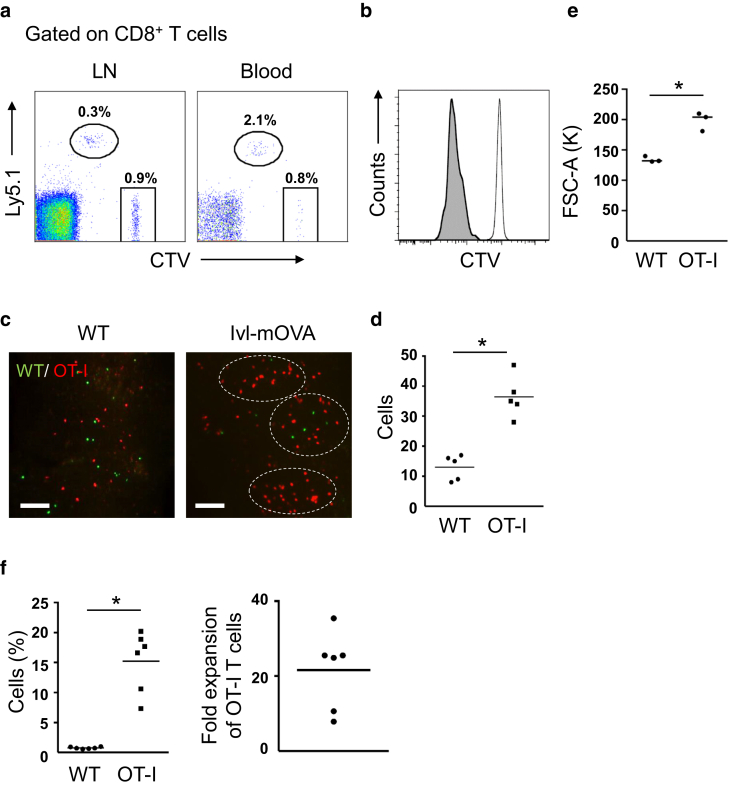
**Transferred OT-I T cells expanded in the skin-draining LNs in an antigen-specific manner.** (**a, b, e**) Flow cytometric analysis 48 hours after the T-cell transfer. (**a**) Representative FACS plots gated on CD8^+^ T cells. WT CD8^+^ T cells (Ly5.2) are shown in a square and OT-I T cells (Ly5.1) are shown in a circle. Histogram of (**b**) CTV fluorescent intensity and (**e**) mean fluorescent intensity of FSC-A of each cell population in inguinal LNs (n = 3 mice). WT CD8^+^ T cells are shown in nontinted area and OT-I T cells are shown in gray tinted area. ∗*P* < 0.05 (*t*-test). Gating strategy for OT-I T cells and WT CD8^+^ T cells in the skin-draining LNs is shown in Figure 3. (**c**) Section view of inguinal LNs 48 hours after the T-cell transfer. White outlined areas indicate the clusters of OT-I T cells (red) in Ivl-mOVA mice. Note that both WT CD8^+^ T cells (green) and OT-I T cells scattered randomly when they were transferred into WT mice. Bar = 200 μm. (**d**) The number of OT-I T cells (red) and WT CD8^+^ T cells (green) from five different microscopic fields of view. ∗*P* < 0.05 (*t*-test). (**f**) Flow cytometric analysis 6 days after the T-cell transfer. Frequency of WT CD8^+^ T cells and OT-I T cells in inguinal LNs (n = 3 mice). Fold expansion is indicated as the ratio of OT-I T cells to WT CD8^+^ T cells. ∗*P* < 0.05 (*t*-test). OT-I T cells indicate CD8^+^ T cells from OT-I mice. CTV, CellTrace Violet; FSC-A, forward scatter area; Ivl, involucrin; K, thousand; LN, lymph node; mOVA, membrane-bound ovalbumin; WT, wild type.

**Figure 3:**
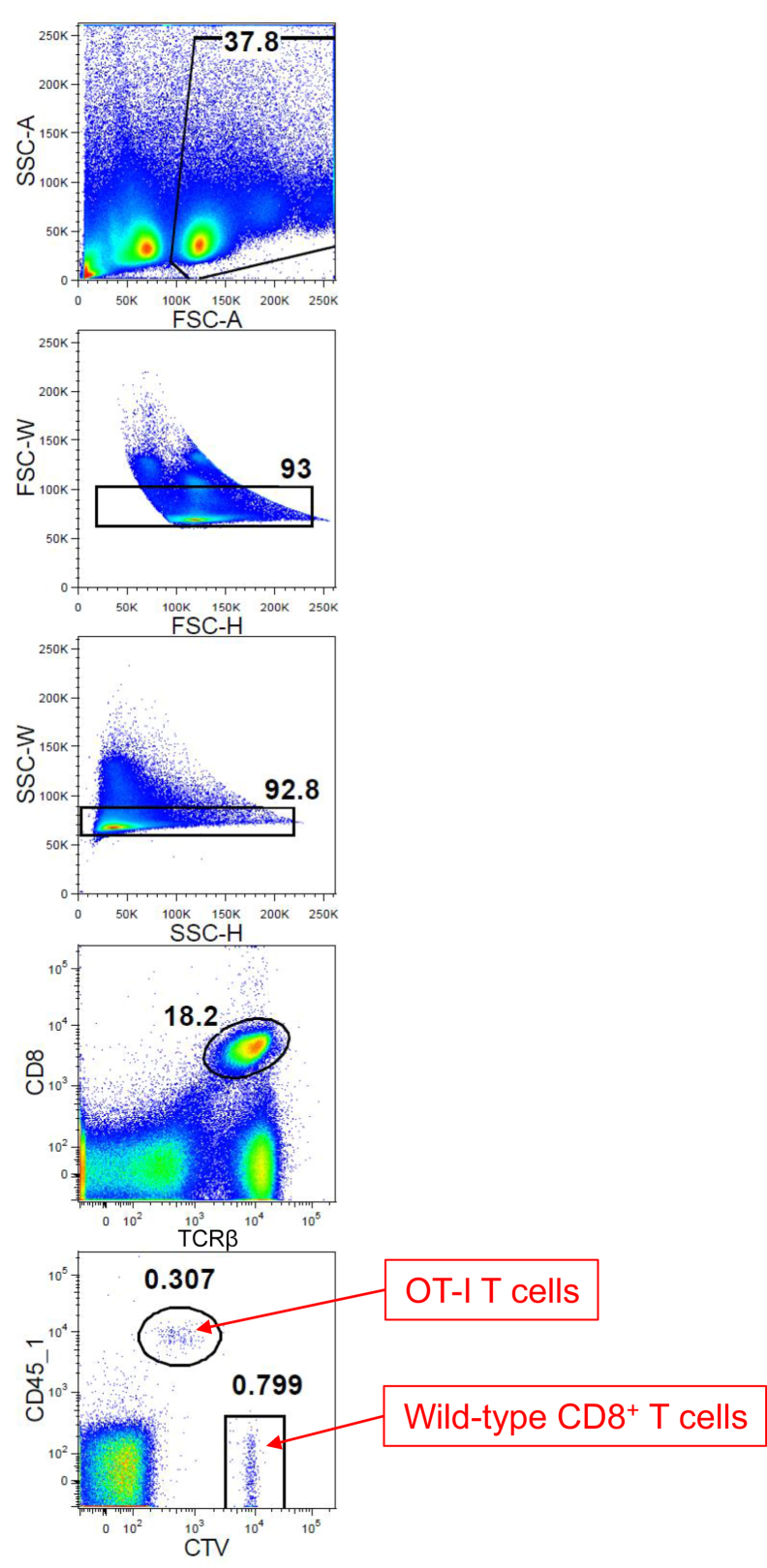
**Gating strategy for OT-I T cells and wild-type CD8^+^ T cells in the skin-draining lymph nodes.** OT-I T cells indicate CD8^+^ T cells from OT-I mice. CTV, CellTrace Violet; FSC-A, forward scatter area; FSC-H, forward scatter height; FSC-W, forward scatter width; K, thousand; SSC-A, side scatter area; SSC-H, side scatter height; SSC-W, side scatter width

To determine whether transferred OT-I T cells expand in Ivl-mOVA mice, we sorted CD8^+^ T cells from Ly5.1^+^ OT-I mice and labeled them with CellTrace Violet, and adoptively transferred to Ly5.2^+^ Ivl-mOVA mice through the tail vein. As a control, we simultaneously transferred the same number of CellTrace Violet–labeled CD8^+^ T cells from Ly5.2^+^ wild-type mice. Forty-eight hours after the T-cell transfer, flow cytometric analysis revealed spontaneous expansion of OT-I T cells but not of wild-type CD8^+^ T cells, in the skin-draining LNs and blood (Figures 2a and b, 3). At this time point, no OT-I T cells were detected in the skin (data not shown). OT-I T cells did not expand when they were transferred into wild-type mice (data not shown).

To examine the distribution and morphological change of OT-I T cells, we harvested the inguinal LNs and observed by a light-sheet fluorescence microscope 48 hours after the simultaneous transfer with wild-type CD8^+^ T cells. OT-I T cells labeled with a red fluorescent protein tdTomato presented larger morphology than EGFP-labeled wild-type CD8^+^ T cells and expanded in clusters (Figure 2c and d). This enlargement of T cells was confirmed by the increased mean fluorescent intensity of forward scatter by flow cytometry (Figure 2e). Six days after the T-cell transfer, flow cytometric analysis revealed that the frequency of OT-I T cells was about 20 times higher than that of wild-type CD8^+^ T cells (Figure 2f). These results show that transferred OT-I T cells spontaneously expand in Ivl-mOVA mice in an antigen-specific manner.

### GVHD-like skin phenotype was induced by the transfer of OT-I T cells

**Figure 4:**
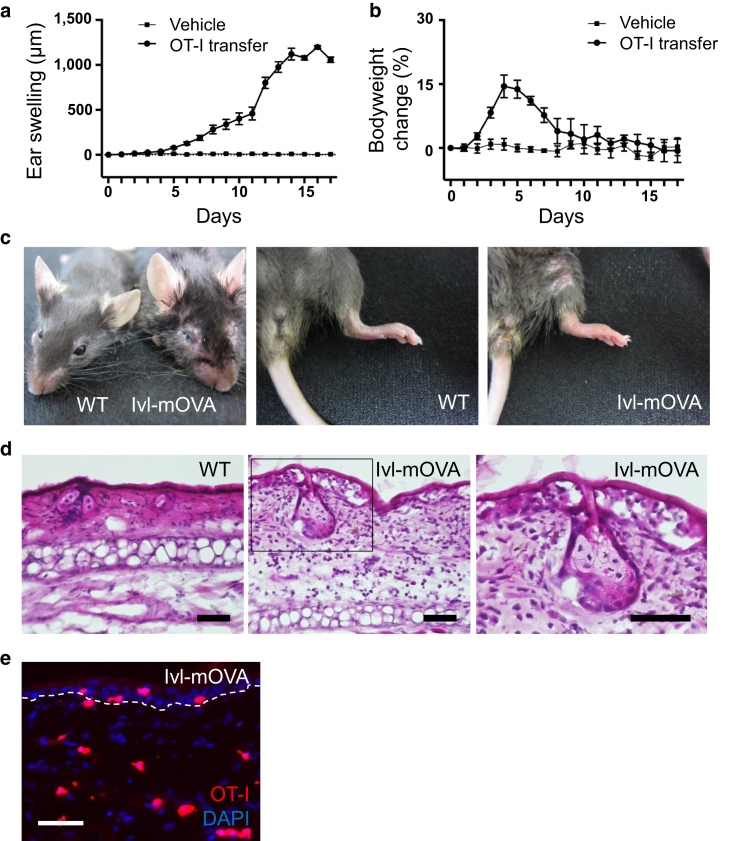
**GVHD-like cutaneous lesions developed after the transfer of OT-I T cells.** (**a**) Ear swelling and (**b**) bodyweight change after the transfer of OT-I T cells (OT-I T cell-transferred group [n = 5 mice] and vehicle-injected group [n = 3 mice], respectively). (**c**) Skin phenotype of Ivl-mOVA mice 10 days after the transfer of OT-I T cells. Head (left panel) and lower limb (middle and right panels). (**d**) H&E staining of the ear skin 14 days after the transfer of OT-I T cells. Right panel is a higher magnification view of the square. Bar = 50 μm. (**e**) Fluorescence microscopy of the ear skin 14 days after the transfer of tdTomato^+^ OT-I T cells. Red, OT-I T cells and blue, DAPI. Bar = 50 μm. OT-I T cells indicate CD8^+^ T cells from OT-I mice. GVHD, graft-versus-host disease; Ivl, involucrin; mOVA, membrane-bound ovalbumin; WT, wild type.

Previous studies using K5/K14-mOVA mice reported that no significant inflammatory skin lesion was developed spontaneously after the transfer of OT-I T cells, except for one K14-mOVA strain, which showed bodyweight loss along with skin inflammation (Azukizawa et al., 2003; Bianchi et al., 2009; Bursch et al., 2009; Miyagawa et al., 2008; Shibaki et al., 2004). Therefore, we monitored body weight and ear thickness as an indicator of skin inflammation in Ivl-mOVA mice after the transfer of OT-I T cells. The ears started to swell five to seven days after the OT-I T-cell transfer, peaked around day 14, and remitted thereafter (Figure 4a). During this observation, Ivl-mOVA mice did not show any weight loss (Figure 4b), suggesting the absence of intake loss and esophageal involvement. Although Ivl is expressed not only in the epidermis but also in other stratified squamous epithelia as with K5/K14, *Ivl* and *mOVA* mRNA expressions were hardly detectable in the esophagus of Ivl-mOVA mice (Figure 1c).

Seven to ten days after the transfer of OT-I T cells to the Ivl-mOVA mice, scaly erythema became apparent in the whole body, especially around the eyes, and the cornea turned nebulous (Figure 4c, left panel). The swelling of footpad was observed as well (Figure 4c, middle and right panels). Histological analysis revealed an infiltration of inflammatory cells into the skin and necrosis of epidermal KC in Ivl-mOVA mice but not in wild-type control mice (Figure 4d). These macroscopical and histological phenotypes were analogous to those found in human GVHD and previous mouse cutaneous GVHD models. Immunohistochemical analysis showed an infiltration of epidermotropic OT-I T cells (Figure 4e). These results indicate that transferred OT-I T cells spontaneously develop GVHD-like skin lesion in Ivl-mOVA mice, without the priming of inflammatory stimuli to the skin and significant bodyweight loss.

### Skin-infiltrated OT-I T cells produced IFN-γ

**Figure 5:**
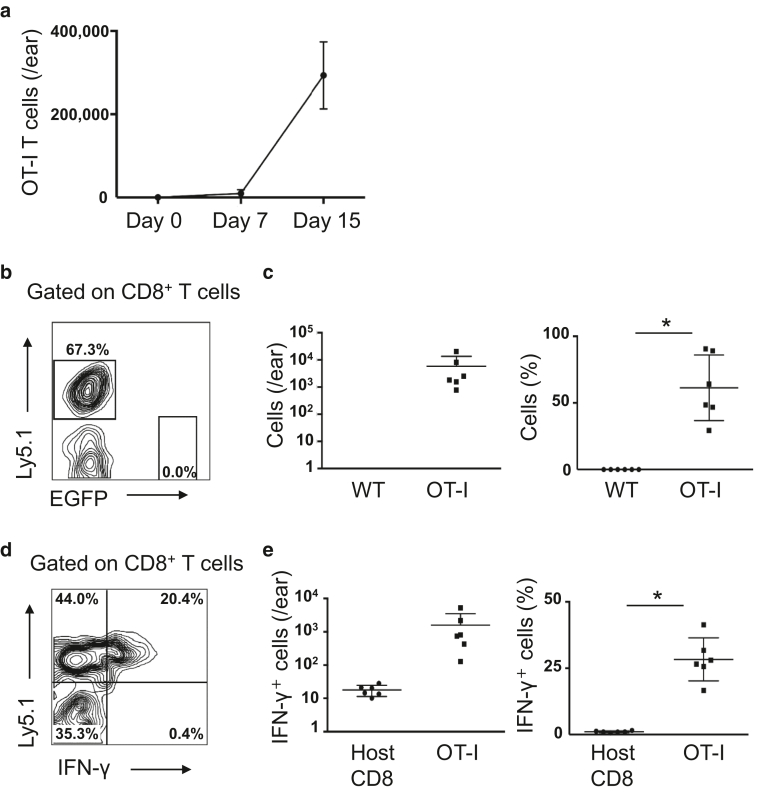
**OT-I T cells infiltrated into the skin and produced IFN-γ.** (**a**) The number of OT-I T cells in the skin during the development of skin inflammation (n = 3 mice). (**b–e**) Flow cytometric analysis of the ear skin 9 days after the T-cell transfer. (**b, d**) Representative FACS plots gated on CD8^+^ T cells. (**c, e**) Quantification (left) and frequency (right) of (**c**) OT-I T-cell infiltration into the skin and (**e**) IFN-γ–producing cells (n = 6 ears). ∗*P* < 0.05 (*t*-test). Gating strategy for IFN-γ–producing OT-I T cells in the skin is shown in Figure 6. OT-I T cells indicate CD8^+^ T cells from OT-I mice. WT, wild type.

To examine the number of OT-I T cells in the skin, we harvested the ears, digested them to single-cell suspensions, and analyzed by flow cytometry. We found that the number of OT-I T cells progressively increased in the skin during the development of skin inflammation (Figure 5a).

**Figure 6:**
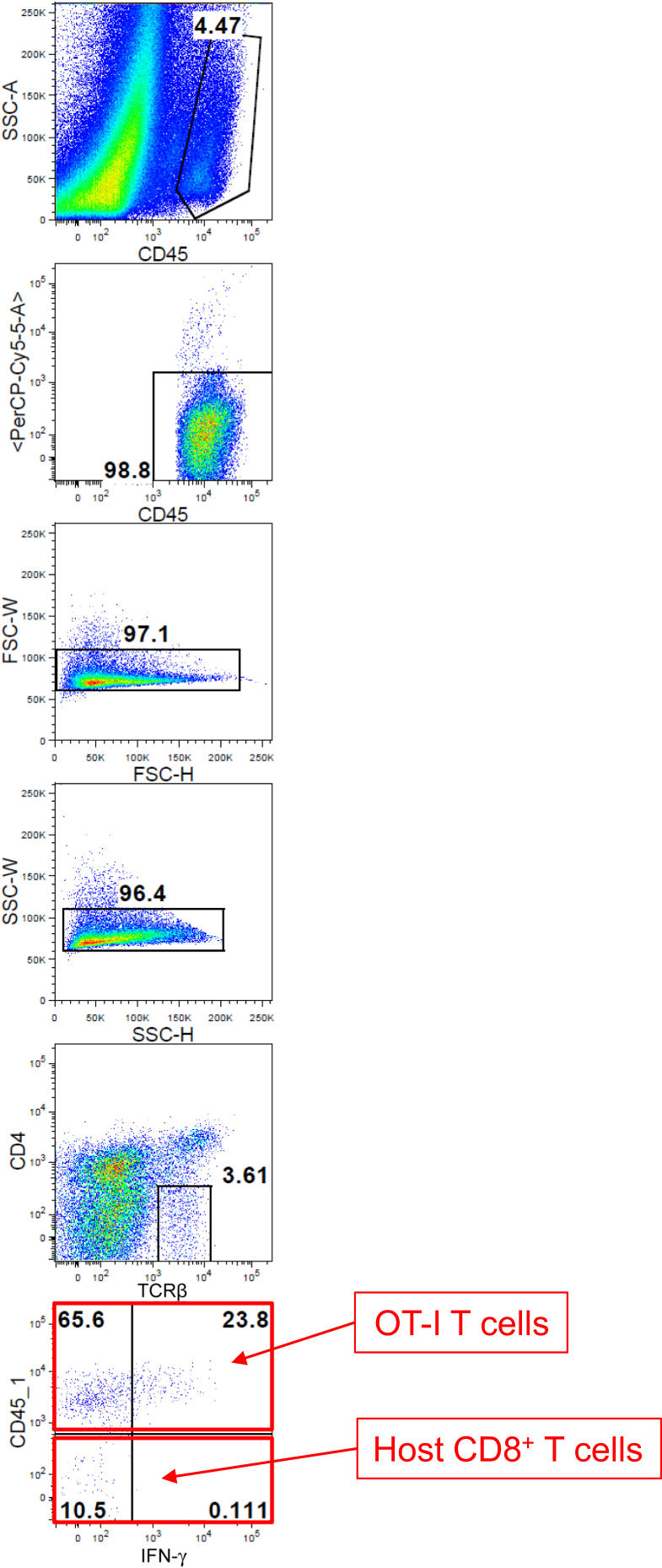
**Gating strategy for IFN-γ–producing OT-I T cells in the skin.** OT-I T cells indicate CD8^+^ T cells from OT-I mice. FSC-H, forward scatter height; FSC-W, forward scatter width; K, thousand; SSC-A, side scatter area; SSC-H, side scatter height; SSC-W, side scatter width.

In the pathogenesis of GVHD, allogeneic CD8^+^ CTLs are the major effector immune cells (Okiyama and Fujimoto, 2015), and IFN-γ produced from activated CTLs is known as a key cytokine (de Araújo-Souza et al., 2015). To elucidate the kinetics of IFN-γ production in the skin, we co-transferred the same number of Ly5.1^+^ OT-I T cells and Ly5.2^+^ EGFP^+^ wild-type CD8^+^ T cells to Ivl-mOVA mice. Nine days after the transfer, a large number of OT-I T cells, but not wild-type CD8^+^ T cells, were detected in the skin at this time point (Figure 5b and c). Approximately one third of the skin-infiltrated OT-I T cells, but no host CD8^+^ T cells in the skin, produced IFN-γ (Figures 5d and e, 6). These results show that the skin-infiltrated OT-I T cells are activated to produce IFN-γ in Ivl-mOVA mice.

**Figure 7:**
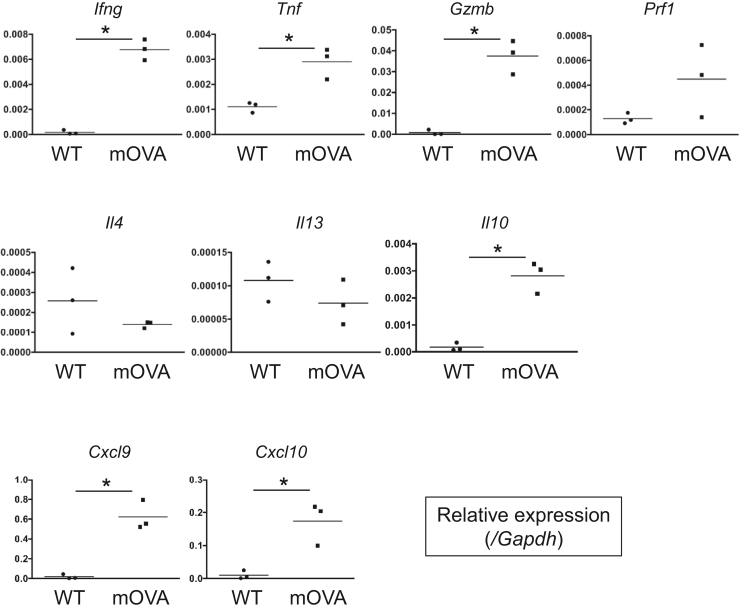
**Quantitative RT-PCR analysis of the ear skin 10 days after the OT-I T-cell transfer.** OT-I T cells indicate CD8^+^ T cells from OT-I mice. ∗*P* < 0.05 (*t*-test). mOVA, membrane-bound ovalbumin; WT, wild type.

Ten days after the OT-I T-cell transfer, we harvested mRNA from the ear skin. Quantitative RT-PCR analysis indicated the cytolytical activity of OT-I T cells and a skewed T-cell balance toward T helper type 1 or Treg, but not T helper type 2 (Figure 7).

### OT-I T cells accumulated around the hair follicles in the early phase of skin inflammation

We sought to investigate when and where OT-I T cells infiltrated into the dermis and subsequently into the epidermis to initiate autoimmune responses in the skin. To achieve this, we transferred tdTomato^+^ OT-I T cells to Ivl-mOVA mice and observed the whole ear skin using a light-sheet fluorescent microscope. After the transfer of OT-I T cells, we harvested the ears, made them transparent by the CUBIC method (Susaki et al., 2014), and stained with Alexa Fluor 488-conjugated anti-CD49f antibody to label epidermal KCs.

**Figure 8:**
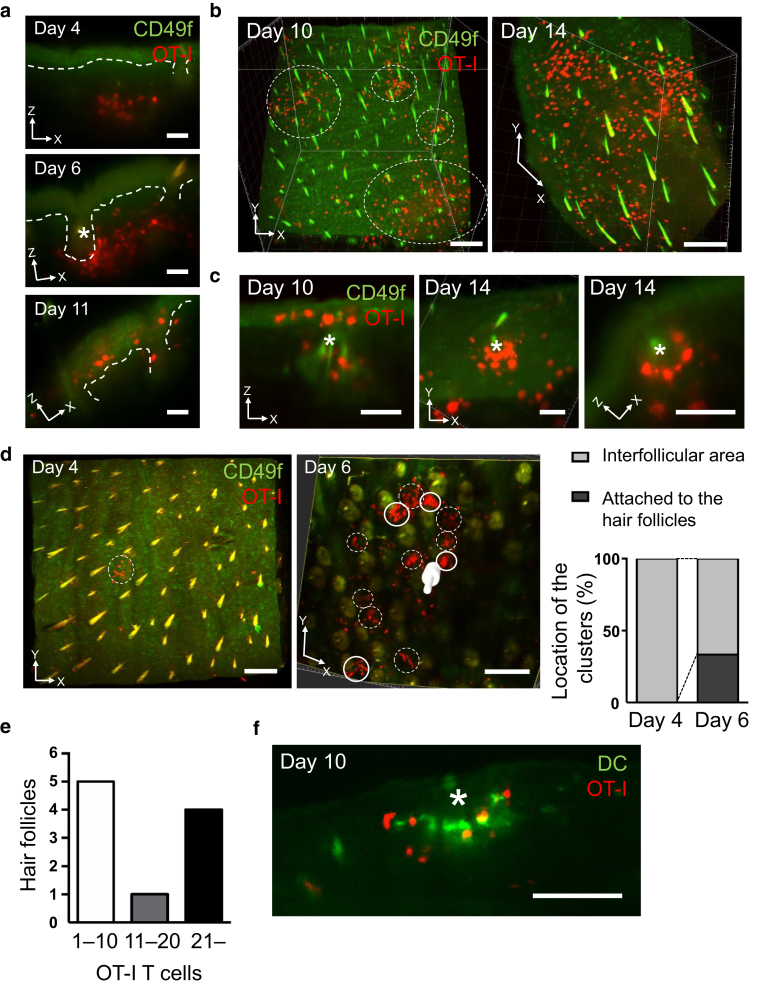
**OT-I T cells accumulated around the hair follicles in GVHD-like skin lesion.** Section view (**a**) and 3D view (**b**) of the ear skin after the transfer of tdTomato^+^ OT-I T cells. White outlined areas indicate clusters of skin-infiltrated OT-I T cells. (**c**) The accumulation of OT-I T cells around the hair follicles. Section view (left and right panels) and 3D view (middle panel). (**d**) Changes in numbers and distribution of OT-I T-cell clusters in the ear skin. Clusters in the interfollicular area (dotted circle) and clusters located in the vicinity of the hair follicles (solid circle) are shown. ∗, hair follicles; red, OT-I T cells; and green, CD49f. Bar = 50 μm (**a** and **c**) and 200 μm (**b** and **d**). (**e**) The number of hair follicles by the number of infiltrated OT-I T cells (out of 60 follicles in the sample of day 6). (**f**) Section view of the ear skin after the transfer of tdTomato^+^ OT-I T cells. ∗, hair follicles; red, OT-I T cells; and green, CD11c^+^ DCs. Bar = 100 μm. OT-I T cells indicate CD8^+^ T cells from OT-I mice. 3D, three dimensional; DC, dendritic cell; GVHD, graft-versus-host disease

OT-I T cells initially appeared in the skin approximately four days after the OT-I T-cell transfer (day 4) (Figure 8a). At this time point, OT-I T cells formed clusters in the dermis, and around day 6, some of them accumulated around the hair follicles (Figure 8a, asterisk). The clusters became larger day by day (Figure 8b, left panel), and diffuse epidermal infiltrations of OT-I T cells were observed around day 10–11 (Figure 8a–c). The accumulation of OT-I T cells around the hair follicles was sustained until day 14 (Figure 8c). Although these observations were not consecutive analysis in the same mouse, only one cluster was detected in the interfollicular area on day 4, whereas one third of clusters were located in the vicinity of the hair follicles on day 6 (Figure 8d and e). These findings suggest that the hair follicles have the potency to attract activated CTLs and may act as portal sites for CTLs to enter the epidermis. Another possibility is that hair follicles attract OT-I T cells through high expression levels of Ivl.

We have previously reported that T cells form clusters with dermal dendritic cells (DCs) for their efficient activation in the skin at the elicitation phase of a mouse contact hypersensitivity model (Natsuaki et al., 2014). Therefore, we wondered if there was also an interplay between dermal DCs and T cells in GVHD-like skin lesion in Ivl-mOVA mice. We crossed Ivl-mOVA mice with CD11c-YFP mice, which express YFP under the control of common DC marker CD11c, and then transferred tdTomato^+^ OT-I T cells into the mice. We observed clusters of YFP^+^ dermal DCs and tdTomato^+^ skin-infiltrated OT-I T cells adjacent to the hair follicles on day 10 (Figure 8f), suggesting the interaction of DCs and T cells in the skin.

### Regulatory T cells increased in the remission phase of GVHD-like skin lesion

**Figure 9:**
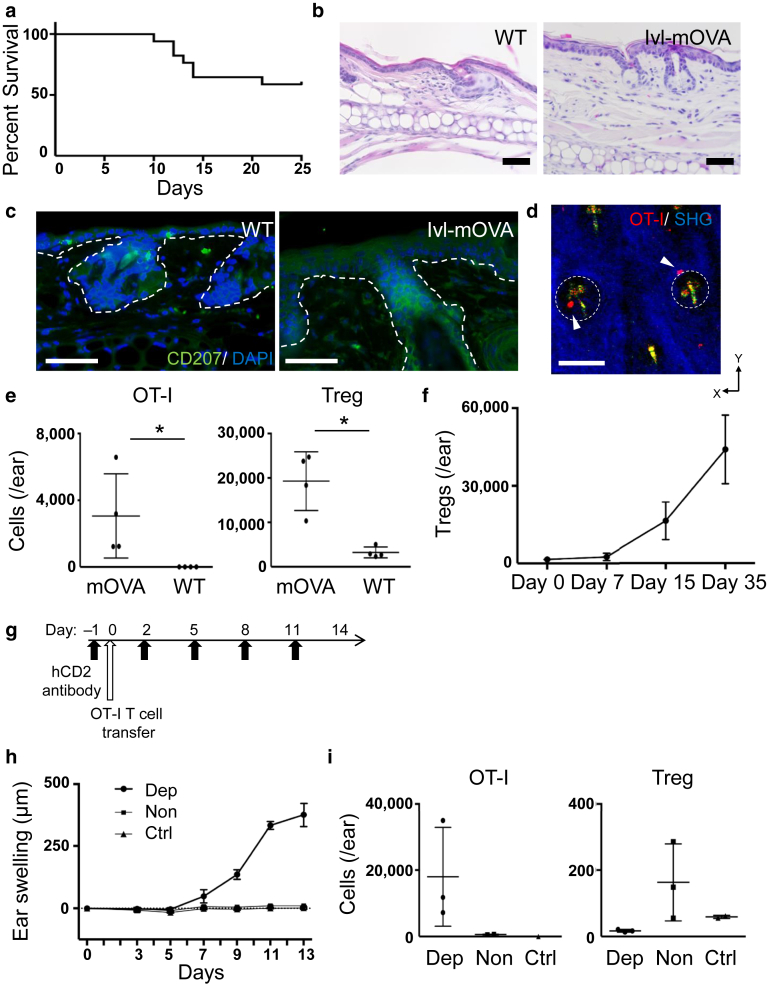
**Survival rate after the transfer of OT-I T cells and the increase of skin-infiltrated Tregs in the remission phase.** (**a**) Survival rate after the transfer of OT-I T cells (n = 17 mice, pooled from three experiments). (**b**) Histological sections (H&E stain) and (**c**) immunohistochemistry of the ear skin 35 days after the transfer of OT-I T cells. Green, CD207 and blue, DAPI. Bar = 50 μm. (**d**) Intravital imaging of the ear skin 35 days after the transfer of tdTomato^+^ OT-I T cells. White outlined areas indicate the hair follicles and arrowheads indicate remaining OT-I T cells inside or in the vicinity of the hair follicles. Red, OT-I T cells; green, autofluorescence; blue, SHG. Bar = 100 μm. (**e**) Flow cytometric analysis of the ear skin 38 days after the OT-I T-cell transfer. Quantification of OT-I T cells (left) and Tregs (right) (n = 4 ears per group). ∗*P* < 0.05 (*t*-test). Gating strategy for Tregs in the skin is shown in Figure 10. (**f**) The number of Tregs in the skin during skin inflammation (n = 3 mice). (**g**) Strategy for in vivo depletion of Foxp3^+^ Tregs by administrating anti-hCD2 antibody. (**h**) Ear swelling after the transfer of OT-I T cells (dep [n = 4 mice], non [n = 4 mice], and ctrl [n = 3 mice]). (**i**) Flow cytometric analysis of the ear skin after the OT-I T-cell transfer. Quantification of OT-I T cells on day 14 (left) and Tregs in the epidermis on day 5 (right) (n = 3 mice except for vehicle-injected control). OT-I T cells indicate CD8^+^ T cells from OT-I mice. Ctrl, vehicle-injected control; dep, Treg-depleted group; hCD2, human CD2; Ivl, involucrin; mOVA, membrane-bound ovalbumin; non, non-Treg-depleted group; SHG, second-harmonic generation; Treg, regulatory T cell; WT, wild type.

Finally, we evaluated the clinical course of GVHD-like skin lesion in Ivl-mOVA mice. We found that approximately half of the Ivl-mOVA mice died within 14 days after the transfer of OT-I T cells (Figure 9a), whereas the remaining half survived for months and the skin inflammation resolved spontaneously. Although we do not have any data on what determines whether each mouse lives or dies, technical variables such as cell viability, injection technique, and mice body weight would influence the survival rate. Histological analysis after the resolution of inflammation revealed a slightly thickened dermis, but no residual epidermal changes were observed (Figure 9b). In human GVHD, it is known that Langerhans cells (LCs) disappear in the epidermis. Consistent with this finding, immunohistochemical analysis showed that CD207^+^ LCs disappeared in the epidermis in Ivl-mOVA mice (Figure 9c).

**Figure 10:**
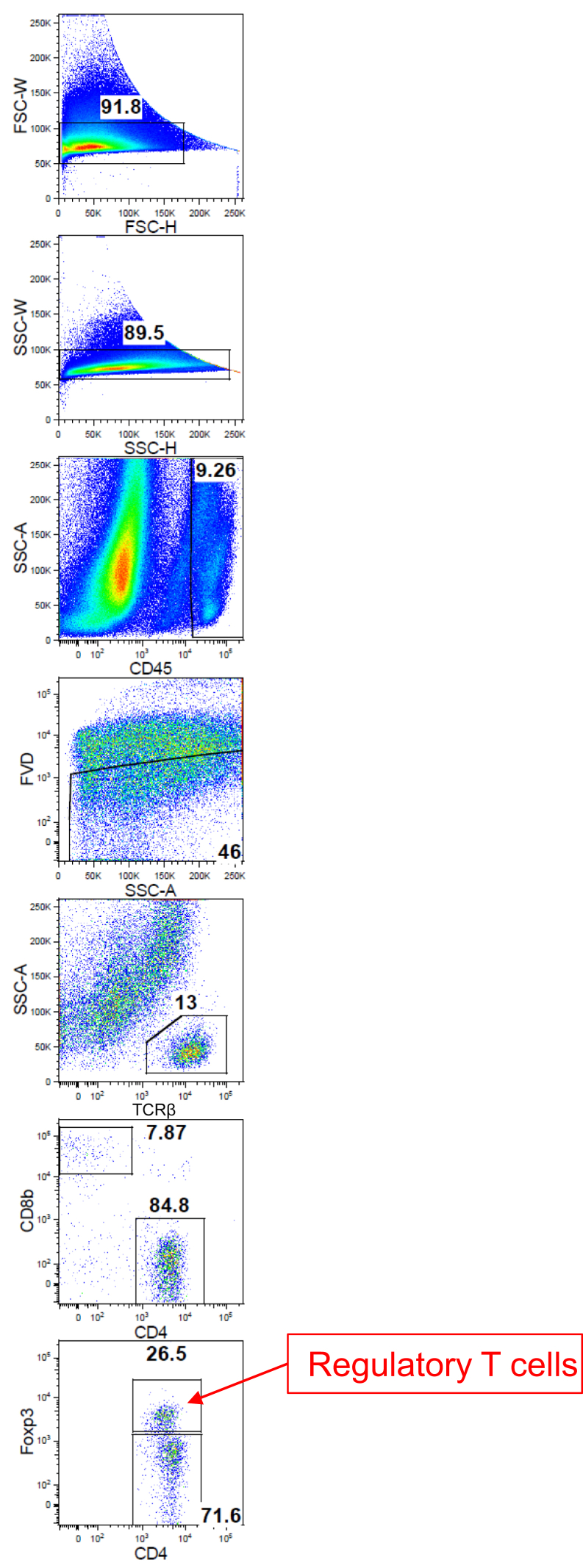
**Gating strategy for regulatory T cells in the skin.** FSC-H, forward scatter height; FSC-W, forward scatter width; FVD, fixable viability dye; K, thousand; SSC-A, side scatter area; SSC-H, side scatter height; SSC-W, side scatter width.

We examined the number of OT-I T cells as well as Tregs in the skin, because it has been reported that Tregs played a pivotal role in the prevention of GVHD in humans and several mouse models (Edinger and Hoffmann, 2011; Ramlal and Hildebrandt, 2017; Riegel et al., 2020; Whangbo et al., 2020). We transferred tdTomato^+^ OT-I T cells into Ivl-mOVA mice, and after the resolution of skin inflammation (30–40 days after the transfer), we examined the number and distribution of OT-I T cells and/or Tregs in the skin by a multiphoton microscope and flow cytometry. Multiphoton microscopic analysis showed remaining OT-I T cells in the skin, and some of them were located inside and in the vicinity of the hair follicles (Figure 9d, arrowheads). Flow cytometric analysis revealed that not only the number of OT-I T cells but also Tregs significantly increased in the skin at the remission phase compared with littermate control which received OT-I T cells (Figures 9e, 10). Particularly, the number of skin-infiltrated Tregs increased during the remission phase (day 15 and after) (Figure 9f).

To further investigate the role of Tregs in vivo, we crossed Ivl-mOVA mice with Foxp3^hCD2^ mice, in which Foxp3^+^ T cells express a glycosylphosphatidylinositol-anchored human CD2–CD52 fusion protein on their cell surface. Using this strain, we can deplete Foxp3^+^ T cells by intravenous administration of anti-human CD2 antibody. We depleted Foxp3^+^ Tregs by administrating anti-human CD2 antibody every 3 days from the day before the transfer (Figure 9g) and transferred 1 × 10^5^ OT-I T cells that were not sufficient to induce skin lesion by themselves. Treg-depleted mice, but not non-Treg-depleted mice, developed skin lesion and showed marked ear swelling (Figure 9h). Flow cytometric analysis clearly revealed the infiltration of OT-I T cells and the depletion of Tregs only in the antibody-administrated mice (Figure 9i).

Taken together, the skin inflammation spontaneously resolved despite remaining OT-I T cells in the skin with increasing skin-infiltrated Tregs and the transfer of a small number (1 × 10^5^) of OT-I T cells could induce skin lesion only in Treg-depleted mice. Although our data do not directly show the role in the resolution of skin inflammation, these results suggest that Tregs suppress the activation of OT-I T cells both in the induction and remission phase of GVHD-like skin inflammation in Ivl-mOVA mice.

## Discussion

In this study, we established a Tg strain, Ivl-mOVA mice, which expressed mOVA as an epidermis-specific self-antigen. After adoptive transfer of OT-I T cells, these mice showed antigen-specific expansion of OT-I T cells in skin-draining LNs, which resulted in spontaneous development of GVHD-like skin lesion without significant bodyweight loss. This skin lesion spontaneously resolved, despite remaining OT-I T cells in the skin with increasing skin-infiltrated Tregs. A caveat of our model is that Ivl-mOVA mice express mOVA predominantly in the suprabasal layers and OT-I T cells attack the epidermis in a major histocompatibility complex class I-restricted manner, whereas cutaneous GVHD is driven by HLA mismatch and presents interface dermatitis affecting primarily the basal cell layer.

Ivl-mOVA mice showed spontaneous development of skin inflammation without bodyweight loss, which was not observed in the previously established Tg strains (Azukizawa et al., 2003; Bianchi et al., 2009; Bursch et al., 2009; Miyagawa et al., 2008; Shibaki et al., 2004). Although the detailed mechanism that induced this phenotype in Ivl-mOVA mice remains unclear, we speculate several possible mechanisms as follows: first, the constructs used to generate mOVA fusion proteins may partly account for these phenotypes. Our Ivl-mOVA mice express OVA fused to PDGF receptor to provide membrane localization as previously reported two K14-mOVA strains (Miyagawa et al., 2008; Shibaki et al., 2004). On the contrary, K5-mOVA mice and other K14-mOVA strains express OVA fused to transferrin receptor (Azukizawa et al., 2003; Bianchi et al., 2009; Bursch et al., 2009). Second, the expression level of the transgene, distinct promoters (K5, K14, and Ivl), and different transgene integration loci may also influence the immune responses. Even among the strains with the same transgene, the phenotype is different. Shibaki et al. developed two K14-mOVA strains and reported that one strain exhibited spontaneous induction of skin inflammation, whereas the other strain did not (Miyagawa et al., 2008; Shibaki et al., 2004). Of note, the strain without any clinical phenotype expressed a rather high level of OVA-derived peptide–major histocompatibility complex class I complexes (Miyagawa et al., 2010). Third, medullary thymic epithelial cells express tissue-specific self-antigens to delete self-reactive T cells (clonal deletion) (Xing and Hogquist, 2012). The relatively high expression of mOVA in K5/K14-expressing medullary thymic epithelial cells compared with that in our Ivl-mOVA strain may also affect the phenotype. Therefore, K5/K14-mOVA mice harbor a limitation that medullary thymic epithelial cells present OVA-derived peptides and influence on the immune reactions of transferred OT-I T cells.

As we repeated the transfer of OT-I T cells with various cell numbers, we found that there was a threshold for the development of GVHD-like skin lesion. When we transferred 1.5 × 10^6^ OT-I T cells, OT-I T cells expanded in skin-draining LNs and resulted in the development of skin lesion. On the contrary, when we transferred 0.5 × 10^6^ cells, OT-I T cells failed to expand and skin lesion was not induced (data not shown). We hypothesized that some factors, such as Tregs, inhibited the activation of OT-I T cells and that the OT-I T-cell number had to overtake these suppressive factors to develop skin lesion successfully. Consistent with this hypothesis, the transfer of a small number (1 × 10^5^) of OT-I T cells could induce skin lesion after Treg depletion. Rosenblum et al. (2011) previously reported a similar phenotype in another mouse model of inducible mOVA expression in the epidermis. Intriguingly, their model also showed skin inflammation caused by OVA-specific CD4^+^ T cells and spontaneously resolved through Tregs-dependent suppression. Not only Tregs but also exhaustion or deletion of CTLs may be involved in the resolution of skin inflammation.

In human GVHD cases, follicular papules are occasionally seen, and follicular involvement is regarded as a pathological finding that may suggest the diagnosis of acute GVHD (Hong et al., 2005). The limitation of our study is that the observations using whole-mount imaging techniques were not consecutive analysis in the same mouse, but transferred OT-I T cells accumulated around the hair follicles, and they formed clusters with CD11c^+^ DCs adjacent to the hair follicles. In this sense, our model may appropriately mimic the pathological mechanisms of human GVHD. Although we have not determined the surface markers of these CD11c^+^ DCs, it has been reported that CD207^+^ CD103^+^ dermal DCs were often found adjacent to the hair follicles (Bursch et al., 2007) and were the only DC subpopulation that were able to cross-present OVA in K5-mOVA mice (Bedoui et al., 2009; Henri et al., 2010). After the resolution of skin inflammation, LCs disappeared in the epidermis in Ivl-mOVA mice. Recipient LCs were reported to be directly killed by donor T cells (Merad et al., 2004). A recent study showed that LCs inhibited the skin-infiltrated OT-I T cells and induced apoptosis in K14-mOVA mice to negatively regulate mucocutaneous lesions (Kubota et al., 2021). Previous studies showed that Ivl expression from hair follicles is hair cycle-dependent, with a substantial decrease during telogen (Adly and Assaf, 2012). The resolution of skin inflammation may be partially due to a reduction in mOVA expression from hair follicles.

In summary, we have established a murine cutaneous GVHD-like skin inflammation model. OT-I T-cell transfer led to spontaneous development of GVHD-like skin lesion. Our Ivl-mOVA mice could be one of the promising tools to investigate the pathogenesis of skin autoimmunity and the tolerance mechanisms in the skin during the remission phase of GVHD.

## Materials and Methods

### Mice

OT-I (Hogquist et al., 1994), CAG-EGFP (Okabe et al., 1997), Rosa26-tdTomato (Madisen et al., 2010), E2a-Cre (Lakso et al., 1996), CD11c-YFP (Lindquist et al., 2004), and Foxp3^hCD2^ (Miyao et al., 2012) mice have been described previously. We crossed Rosa26-tdTomato with E2a-Cre to generate ubiquitously tdTomato-expressing mice. Offsprings were further crossed with OT-I mice (tdTomato^+^ OT-I mice). All experimental protocols were approved by the Animal Experimentation Committee of Kyoto University (Kyoto, Japan) and all animal experimental procedures were performed according to the Animal Protection Guidelines of Kyoto University.

### Construction of *mOVA* transgenic vector and generation of *mOVA*^*Tg*^ mice

To construct the Ivl-mOVA vector (Figure 1a), a human Ivl promoter vector (pH3700-pL2; provided by Lorne B. Taichman) (Carroll and Taichman, 1992) and a p*K14-mOVA* vector (Shibaki et al., 2004) were digested with NotI (New England Biolabs, Ipswich, MA) and DNA fragment of *mOVA* (1,334 base pairs) was inserted to human Ivl promoter vector by using DNA Ligation Kit (Takara Bio, Kusatsu, Japan). The constructed vector was expanded and linearized with HindIII (New England Biolabs). The purified DNA fragments (8,357 base pairs) were injected into pronuclei of fertilized eggs and implanted into pseudopregnant females by generous assistance from Jun Kudo and Kosuke Sakai, Laboratory of Gene Medicine, Keio University School of Medicine (Tokyo, Japan). The mouse transgene was screened by PCR to amplify the region of SV40 intron (166 base pairs) using 5*′*-AgTCTTTTTgTCTTTTATTTCAggTC-3*′* as a forward primer and 5*′*-AgCCgAATTCCAgCACACT-3*′* as a reverse primer.

### Histology and immunohistochemistry

For histological examination, tissues were fixed with 10% formalin in PBS and were embedded in paraffin. Sections were stained with H&E. For immunohistochemical staining, samples were immersed in 1% paraformaldehyde (Nacalai Tesque, Kyoto, Japan) overnight at 4 °C, embedded in OCT compound (Sakura Finetek, Tokyo, Japan), frozen, and then sectioned. After treatment with Image-iT FX Signal Enhancer (Life Technologies, Palo Alto, CA), sections were incubated with anti-OVA (Polysciences, Warrington, PA) and anti-CD207 (929F3.01, Novus Biologicals, Centennial, CO) antibody overnight at 4 °C, and then with Alexa Fluor 488 anti-rabbit/rat IgG (Life Technologies) for 60 minutes, respectively. As a negative control, we used a sample stained with secondary antibody alone. The slides were mounted using ProLong Antifade with DAPI (Life Technologies). Images were captured on a fluorescence microscope (BZ-900, Keyence, Osaka, Japan).

### Quantitative PCR analysis

Total RNA was isolated using RNeasy Mini Kit (Qiagen, Hilden, Germany). To separate the epidermis and the dermis, dorsal halves of ears were incubated in 0.25% Trypsin and EDTA. cDNA was synthesized using PrimeScript RT reagent kit and random hexamers according to the manufacturer’s protocol (Takara Bio). LightCycler 480 and SYBR Green were used according to the manufacturer’s protocol (Roche, Basel, Switzerland) for quantitative PCR. The expression of each gene was normalized to that of the control gene *Gapdh*. The primers used in this study are available on request.

### Cell sorting and adoptive transfer

CD8^+^ T cells were magnetically isolated using a CD8a^+^ T Cell Isolation Kit and autoMACS according to the manufacturer’s protocol (Miltenyi Biotec, Gladbach, Germany). Unless otherwise stated, 1.0–1.5 × 10^6^ cells were injected into each group of mice through the tail vein. For cell proliferation assay, sorted CD8^+^ T cells were labeled with CellTrace Violet according to the manufacturer’s protocol (Invitrogen, Carlsbad, CA) before adoptive transfer.

### Antibodies and flow cytometry

Anti-mouse CD45 (30-F11), CD8a (53-6.7), and IFN-γ (XMG1.2) antibodies were purchased from BD Biosciences (Franklin Lakes, NJ); CD45.1 (A20), CD4 (RM4-5), and TCRβ (H57-597) antibodies from BioLegend (San Diego, CA); CD8b (H35-17.2) antibody from eBioscience (San Diego, CA); CD4 (RM4-5) and Foxp3 (FJK-16s) antibodies from Invitrogen. Dorsal halves of ears were digested with Liberase TL (Roche) containing 0.05% DNase I (Sigma-Aldrich, St. Louis, MO) for 60 minutes at 37 °C. The digested tissues were meshed through 40 μm of cell strainer to obtain single-cell suspensions. For intracellular staining, 10 μg/ml brefeldin A (Sigma-Aldrich) was put in the digestion buffer, and then collected cells were fixed and permeabilized with Cytofix/Cytoperm buffer (BD Biosciences). For intranuclear staining, cells were fixed and permeabilized using Transcription Factor Buffer Set (BD Biosciences). Flow cytometry was performed using LSRFortessa (BD Biosciences) and data were analyzed with FlowJo software (Tree Star, Ashland, OR).

### Whole-mount and intravital imaging techniques

For whole ear skin clearing, we employed CUBIC method as described (Susaki et al., 2014) with some modifications. Anti-mouse CD49f (GoH3) antibody was purchased from BioLegend. Images were captured on a light-sheet fluorescence microscope (Z.1, Carl Zeiss, Oberkochen, Germany). For intravital imaging, mice were positioned on the stage of a multiphoton microscope (IX-81, Olympus, Tokyo, Japan) and their ear lobes were fixed on a cover glass with a single drop of immersion oil.

### Depletion of Tregs

For depletion of Tregs, Ivl-mOVA mice were crossed with Foxp3^hCD2^ mice. Anti-human CD2 antibody (Campath-1G) (Hale et al., 1987) was administrated intravenously (200 μg per mouse) every 3 days from the day before the OT-I T-cell transfer.

### Statistical analysis

Unless otherwise indicated, data are presented as means ± SDs and each data point is representative of three independent experiments. Statistical analyses were performed using GraphPad prism (GraphPad Software, San Diego, CA). Normal distribution was assumed a priori for all samples. Unless indicated otherwise, a parametric unpaired two-tailed *t*-test was used for comparing two groups. A value of *P* < 0.05 at 95% confidence intervals was considered to indicate statistical significance.

### Data availability statement

No datasets were generated or analyzed during this study.

## ORCIDs

Yujin Nakagawa: http://orcid.org/0000-0001-7994-0457

Gyohei Egawa: http://orcid.org/0000-0002-6101-4719

Toshiya Miyake: http://orcid.org/0000-0003-3122-4528

Saeko Nakajima: http://orcid.org/0000-0003-0831-1447

Atsushi Otsuka: http://orcid.org/0000-0001-7365-947X

Takashi Nomura: http://orcid.org/0000-0002-4004-1339

Akihiko Kitoh: http://orcid.org/0000-0003-4724-8807

Teruki Dainichi: http://orcid.org/0000-0002-9497-0029

Jun-ichi Sakabe: http://orcid.org/0000-0002-8237-0472

Akihiko Shibaki: http://orcid.org/0000-0001-5159-6092

Yoshiki Tokura: http://orcid.org/0000-0001-7452-6919

Tetsuya Honda: http://orcid.org/0000-0003-2355-4869

Kenji Kabashima: http://orcid.org/0000-0002-0773-0554

## Author Contributions

Conceptualization: YN, GE, TH, KK; Formal Analysis: YN, TM, SN; Funding Acquisition: KK; Investigation: YN, GE, TM, SN; Methodology: YN, GE, SN, JiS, KK; Project Administration: KK; Resources: KK; Supervision: GE, TH, KK; Validation: GE, TH; Visualization: YN; Writing - Original Draft Preparation: YN; Writing - Review and Editing: GE, TH, KK

## Conflict of Interest

The authors state no conflict of interest.
